# Placement of an artificial urethral sphincter in 8 male dogs with urethral diverticulum

**DOI:** 10.1111/jvim.17102

**Published:** 2024-05-15

**Authors:** Geoffrey Neumann, Catherine Vachon, William T.N. Culp, Carrie Palm, Julie K. Byron, Joanna Pogue, Marilyn Dunn

**Affiliations:** ^1^ Department of Clinical Sciences, School of Veterinary Medicine University of Montreal Saint‐Hyacinthe Quebec Canada; ^2^ Department of Veterinary Surgical and Radiological Sciences UC Davis Veterinary Medicine Davis California USA; ^3^ Department of Veterinary Clinical Sciences Ohio State University Columbus Ohio USA

**Keywords:** canine, incontinence, occluder, urinary tract

## Abstract

**Background:**

Urethral diverticulum (UD) is a poorly defined anomaly consisting of an outpouching of the urethra. Management without surgical resection is not previously reported in dogs.

**Hypothesis/Objectives:**

Report the outcome of male dogs presented for urinary incontinence with UD treated with an artificial urethral sphincter (AUS).

**Animals:**

Eight client‐owned dogs with UD treated with an AUS.

**Methods:**

Multicenter retrospective study. Medical records from male dogs with urinary incontinence were reviewed. Inclusion criteria:  diagnosis of a UD by retrograde cystourethrography, cystoscopy, abdominal ultrasonography or contrast computed tomodensitometry (CT) or a combination of these modalities, AUS placement, and at least 1 follow‐up. Urinary continence score (UCS) was attributed retrospectively.

**Results:**

Median UCS at presentation was 1/5. A contrast cystourethrogram was diagnostic in 8/8 dogs. All diverticula were saccular, and 7/8 were within the prostatic urethra and 1/8 extended up to the membranous urethra. A congenital origin was suspected in 7 dogs and acquired in 1. Concurrent anomalies included renal dysplasia or chronic pyelonephritis (n = 4), bilateral cryptorchidism (n = 3), and pelvic urinary bladder (n = 3). All dogs were poorly/moderately responsive to phenylpropanolamine. Artificial urethral sphincter placement resulted in improvement in continence in all dogs with a median UCS of 4/5 (5/5 in 2/8 dogs, 4/5 in 5/8 dogs, 3/5 in 1/8 dogs).

**Conclusion:**

Urethral diverticulum should be considered in male dogs with persistent urinary incontinence not responding to medical management. Artificial urethral sphincter placement is an effective therapeutic option that improved continence scores in all dogs.

**Abbreviations**
AUS: artificial urethral sphincter
CT: computed tomodensitometry
PPA: phenylpropanolamine
UCS: urine continence score
UD: urethral diverticulum

## 1 INTRODUCTION

Urethral diverticulum (UD) is poorly defined in veterinary medicine, with only 2 case reports describing this anomaly in male dogs.^1^, ^2^ In humans, confusion exists regarding the terminology used to describe the numerous variations of UD.^3^ The term UD is used to describe a set of urethral diseases with variable presentations (cysts, urethral dilatations, pouches).^3^, ^4^ Urethral diverticulum might vary in size and location.^5^

In male dogs, urinary incontinence is uncommon and is reported in 1% of male dogs presented to a primary care veterinary practice. The 2 most common causes of incontinence were urethral sphincter mechanism incompetence and ectopic ureter.^6^

The artificial urethral sphincter (AUS) is a surgically placed implant used to treat urinary incontinence in dogs and cats with confirmed diagnosis of urinary sphincter mechanism incompetence that have failed to respond to medical or surgical management.^7^, ^8^, ^9^, ^10^ An AUS is composed of a silicone cuff placed around the urethra and attached to a subcutaneous port that can be infused with sterile water or saline. Infusion of the cuff results in partial mechanical obstruction of the urethra. After AUS placement, improved urine continence scores (UCS) in 60% to 100% of dogs is reported, and the AUS can be infused to adjust for changes in continence in the short and long term.^7^, ^8^, ^11^, ^12^

Placement of an AUS is more commonly performed in female dogs with only 1 retrospective study in male dogs.^7^ Artificial urethral sphincter placement is associated with both minor and major complications in male dogs. Minor complications included stranguria (13%), mild inflammation around the port (6%), and hematoma (6%) that resolved within a few days after surgery.^7^ Urethral obstruction (13%), rotation of the port (6%), and fistula (13%) are considered major complications requiring surgical revision.^7^ Unlike in humans, UD is not reported as a complication of AUS placement in dogs.

The aim of our study was to assess the short‐ and long‐term outcome of AUS placement in male dogs with UD.

## 2 MATERIALS AND METHODS

Medical records of male dogs from the University of Montreal, Centre Hospitalier Universitaire Vétérinaire presenting between February 2009 and August 2023, having undergone AUS placement were reviewed. Recruitment was also performed via email list serves of the following colleges and societies: the American College of Veterinary Internal Medicine, the American College of Veterinary Surgeons, the European College of Veterinary Surgeons, the American Society of Veterinary Nephrology and Urology, and the Veterinary Interventional Radiology and Interventional Endoscopy Society. Inclusion criteria consisted of male dogs presented for urinary incontinence diagnosed with a UD and treated with placement of an AUS. Images, medical reports, or both had to be available for review and be consistent with the diagnosis of a UD. At least 1 postoperative follow‐up (examination or phone communication) was also required. Urethral diverticulum was defined as a congenital or acquired outpouching in continuity with the urethra.

Retrieved data included signalment, timing of AUS placement, preexisting medical conditions and anomalies/concurrent congenital disorders, previous medical or surgical treatments, size of the AUS placed, minor and major complications, number of inflations, and total volume instilled in the cuff. Minor complication is defined as a complication that was successfully treated medically, whereas a major complication is a complication requiring surgical revision. Variables related to the UD, including type of diverticulum (saccular, defined as an outpouching of the urethra forming a saccular defect lined with urethral epithelium in continuity with the urethra by means of a discrete orifice,^3^, ^4^, ^5^ or diffuse) location (prostatic, membranous, and penile), and suspected etiology (congenital or acquired) were recorded.

To assess the severity of urinary incontinence, a UCS was assigned to each dog based on a 5‐point continence score scale (Table 1) at presentation and at each recheck visit.^13^ Based on follow‐up results, the highest UCS after the procedure was recorded for each dog. The lowest score (1/5) indicated continuous urine dribbling; the highest score (5/5) indicated full continence.

**TABLE 1:** Urinary continence scoring system.

Score	Description
1	The dog is constantly leaking urine when laying down and when exercising.
2	The dog is poorly continent and intermittently leaking urine. Urine puddles form when laying down.
3	Occasional urine leaking when exercising only (running, walking, playing).
4	The dog is mostly continent, with occasional urine leak (drops only) only when laying down.
5	Dog is always continent with no urine leakage.

AUS were placed surgically as described, and the size of the occluder was determined according to the size of the urethral circumference measured intraoperatively.^7^, ^12^

## 3 RESULTS

Eight male dogs (5 intact, 3 neutered) from 3 teaching hospitals (University of Montreal, n = 5; University of California, Davis, n = 2; Ohio State University, n = 1) were included in the study. Breeds included West Highland white terrier (n = 1), Bernese mountain dog (n = 1), Great Dane (n = 1), rottweiler (n = 1), terrier mix (n = 1), golden retriever (n = 1), border terrier (n = 1), and standard poodle (n = 1). The median age at diagnosis of the UD was 11 months [range, 4‐58], and the median age at AUS placement was 24 months [range, 8‐72]. The median weight at the time of diagnosis of the UD was 23.6 kg [range, 4.9‐48].

All dogs were initially evaluated for urinary incontinence. Two dogs also presented with a weak urine stream. In 7/8 dogs (87.5%), urinary incontinence was observed or suspected since birth. One dog became incontinent after neutering at 1 year of age. The median UCS at presentation was 1/5 (1/5 [n = 7], 2/5 [n = 1]).

A congenital origin was suspected in 7/8 dogs: 1 dog developed urethral sphincter incompetence after neutering, and a cystoscopy performed at 12 months of age revealed a suspected congenital UD. In another recently adopted dog, diagnosis of UD was made at 3 years of age. The urinary incontinence was present at adoption, but the onset was unknown. The dog was diagnosed with a UD and urethrocolic fistula at the same time and a congenital origin was suspected. In 1 dog, UD was noted after the correction of bilateral ectopic ureter associated with ureteroceles. Correction of the ectopic ureters did not improve the urinary incontinence and led to the formation of a pouch at the previous site of the ureteroceles in the prostatic urethra, and this was considered an acquired UD.

### 3.1 Concurrent anomalies

A urine culture was performed in all dogs before referral. Urine culture was positive in 5/8 dogs (*Escherichia coli*, n = 4; *Enterococcus spp*., n = 1; *Enterococcus faecalis*, n = 1; *Staphylococcus pseudintermedius*, n = 1) and treated based on culture and sensitivity testing. The treatment of the urinary tract infection did not improve UCS in any dog. After AUS placement, 3 urine cultures were repeated (2 in 1 dog and 1 in another dog) and were negative.

Seven dogs were diagnosed with the following concurrent urogenital anomalies: ultrasonographic findings consistent with renal dysplasia or chronic pyelonephritis (n = 4), bilateral cryptorchid (n = 3), pelvic bladder (n = 3), persistent urachus (n = 1), ectopic location (ventrolateral surface at mid bladder level) of the ureterovesicular junctions (n = 1), and urethrocolic fistula (n = 1). The dog that became incontinent after neutering did not have concurrent urogenital anomalies; however, a copper hepatopathy was diagnosed.

### 3.2 Medical treatments

Phenylpropanolamine (PPA) was administered to all dogs before to AUS placement. Doses ranged from 1 to 2 mg/kg PO q8h to q12h for a median duration of treatment of 6 months. No improvement was observed in 3 dogs, and mild to moderate improvement was noted in 5/8 dogs. Two dogs received testosterone cypionate (1.3 mg/kg IM every 4 weeks) and testosterone undecanoate (dosage and route of administration unknown), respectively, in conjunction with PPA without further improvement. Two dogs underwent injection of a urethral bulking agent (Macroplastique Uroplasty, Inc., Minnetonka, Minnesota, USA and VetFoam, BioChange Ltd, Kibuts Nahsholim, Israel), 1 in the proximal urethra and 1 proximal to the os penis with mild and temporary improvement.

### 3.3 Diagnostic imaging

All dogs underwent abdominal ultrasonography and a contrast cystourethrogram. Abdominal ultrasonography did not show evidence of UD in 7/8 dogs. One dog had a tortuous and dilated proximal urethra on ultrasound and underwent contrast computed tomodensitometry (CT) to confirm the presence of a UD. The prostate was not visible on ultrasound in 1/8 dogs, difficult to visualize in 2 dogs, appeared normal in 2 dogs, and not mentioned in the ultrasound report and no images of the prostate were available for review in 3/8 dogs.

**FIGURE 1:**
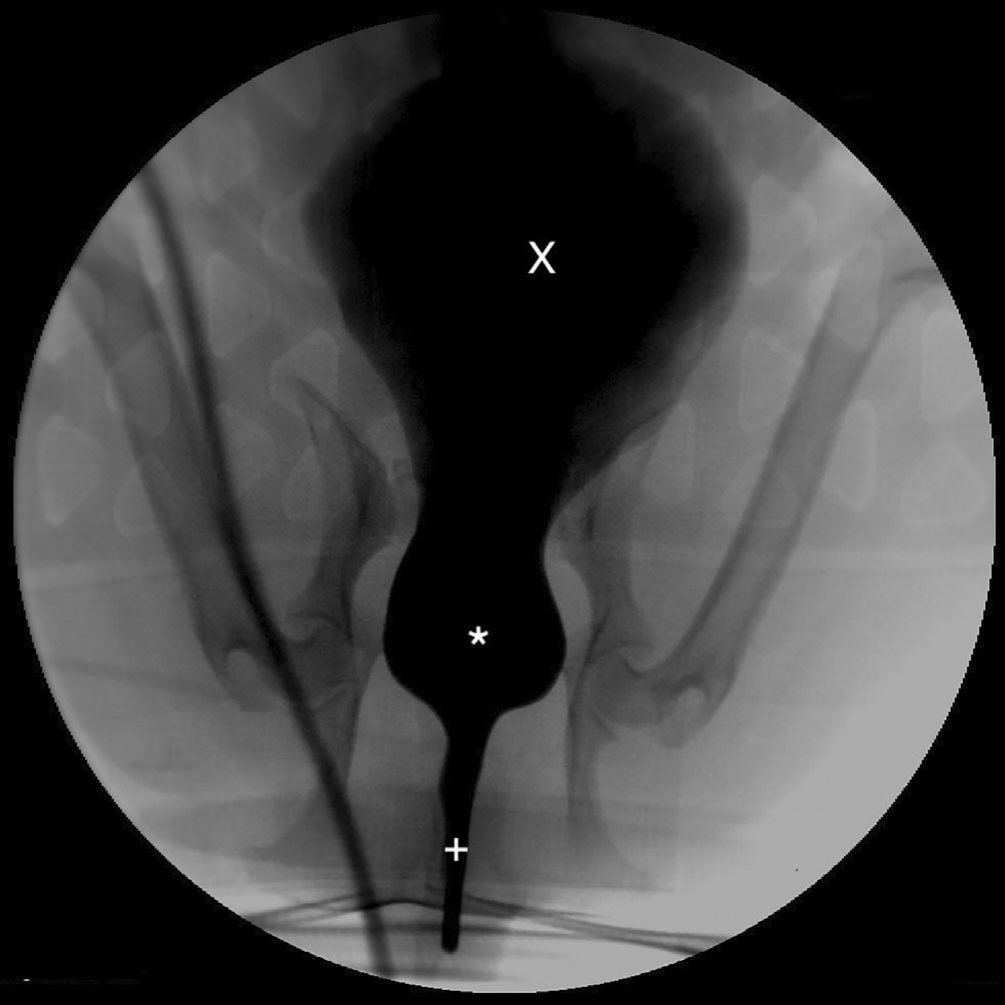
Contrast cystourethrogram performed under fluoroscopic guidance. The dog is placed in dorsal recumbency. A saccular urethral diverticulum is visible in the prostatic urethra (*). Urinary bladder (X), normal urethra (+).

**FIGURE 2:**
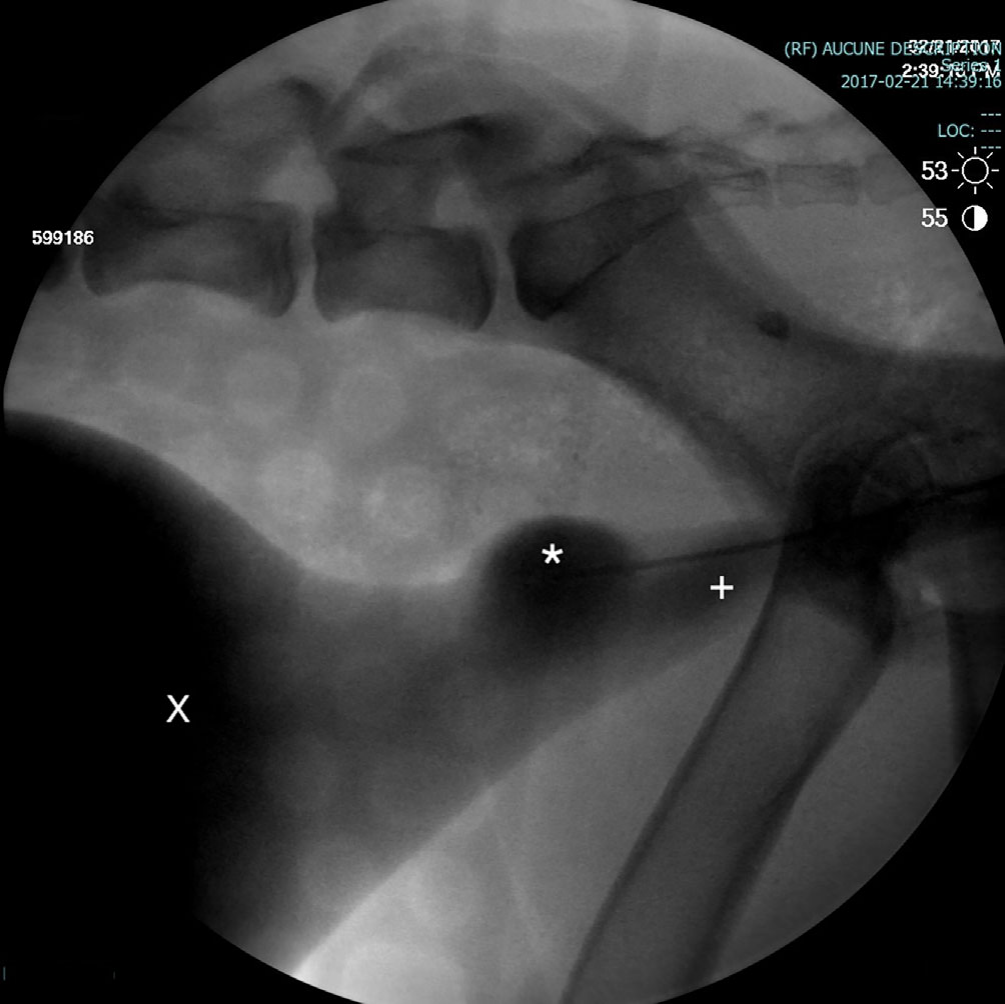
Contrast cystourethrogram performed under fluoroscopic guidance. The dog is placed in lateral recumbency. A saccular urethral diverticulum is visible in the prostatic urethra (*). Urinary bladder (X) enlarged urethra (+).

**FIGURE 3:**
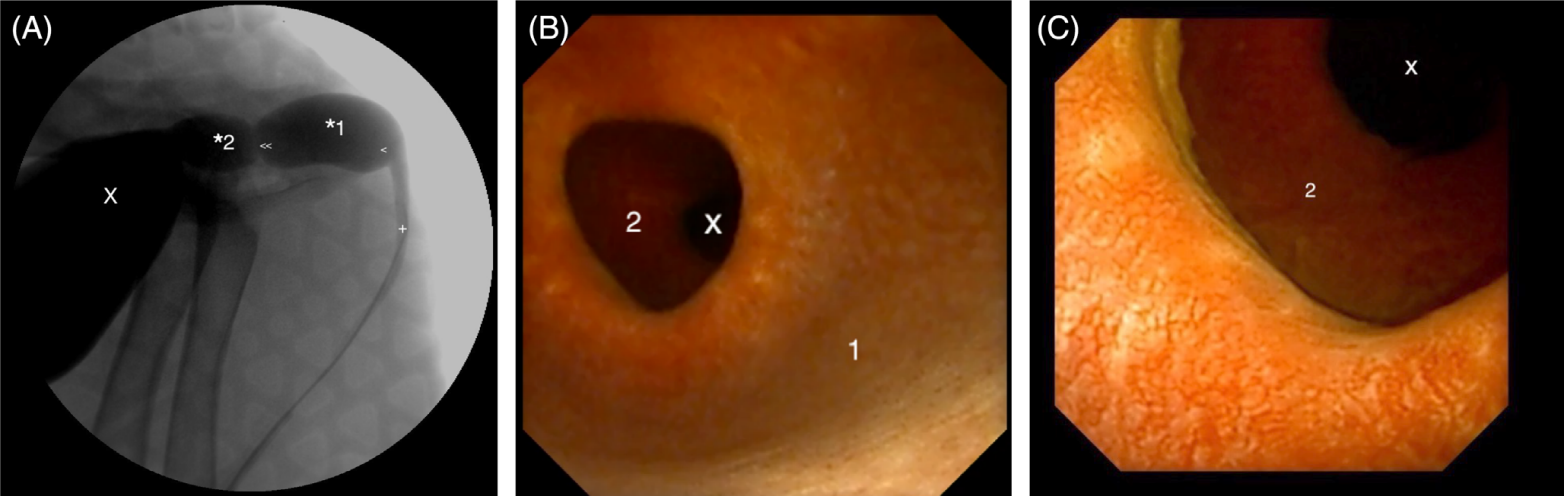
(A) Contrast cystourethrogram performed under fluoroscopic guidance. The dog is placed in lateral recumbency. A bilobed saccular urethral diverticulum is visible in the prostatic urethra (*1: 1st UD, *2: 2nd UD). (<) Location of urethroscope in (B). (<) Location of urethroscope in (C) (<<). Urinary bladder (X), normal urethra (+). (B) Retrograde cystoscopic view of the urethral diverticulum in the same dog as (A). (B) and (C) Retrograde cystoscopic view of the urethral diverticulum in the same dog as (A). Visualization of a large pouch‐like structure within the prostatic urethra (1: 1st UD; 2: 2nd UD), bladder lumen (X).

**FIGURE 4:**
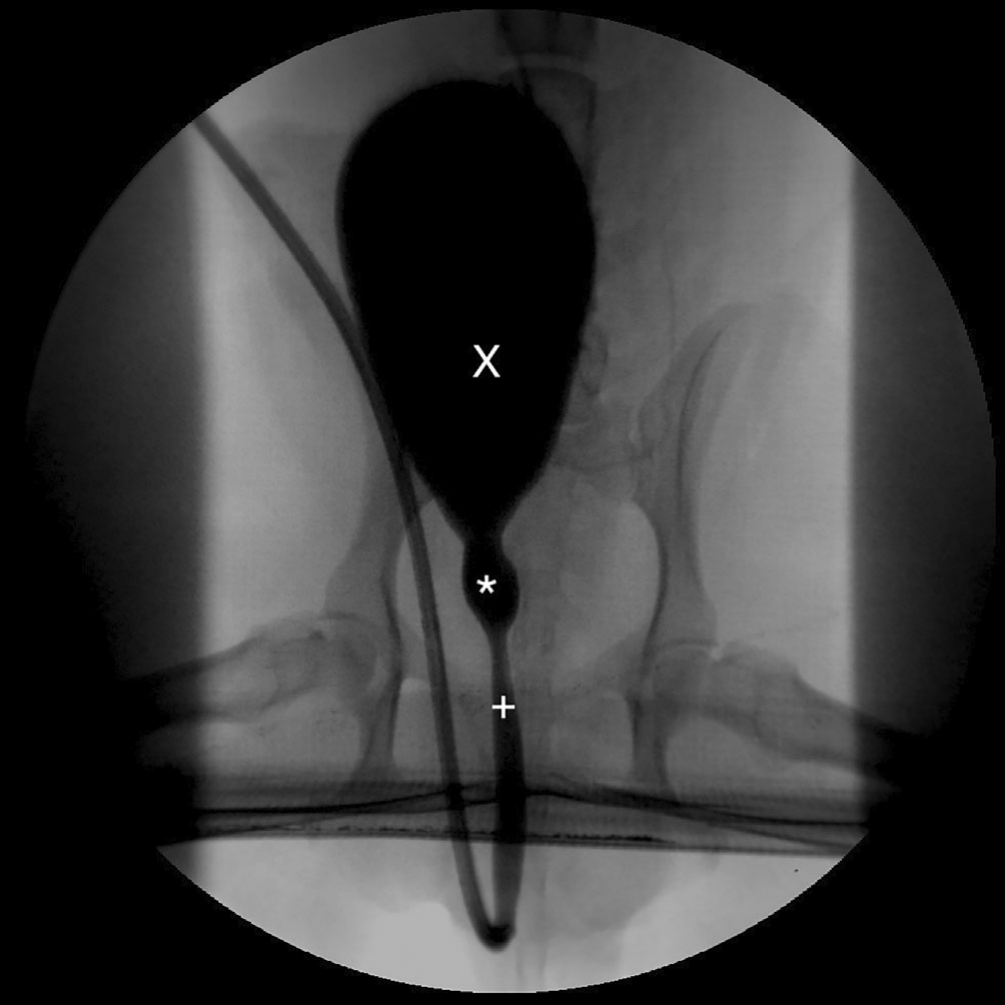
Contrast cystourethrogram performed under fluoroscopic and cystoscopic guidance. The dog is placed in dorsal recumbency. A saccular urethral diverticulum is visible in the prostatic urethra (*). Urinary bladder (X), normal urethra (+).

**FIGURE 5:**
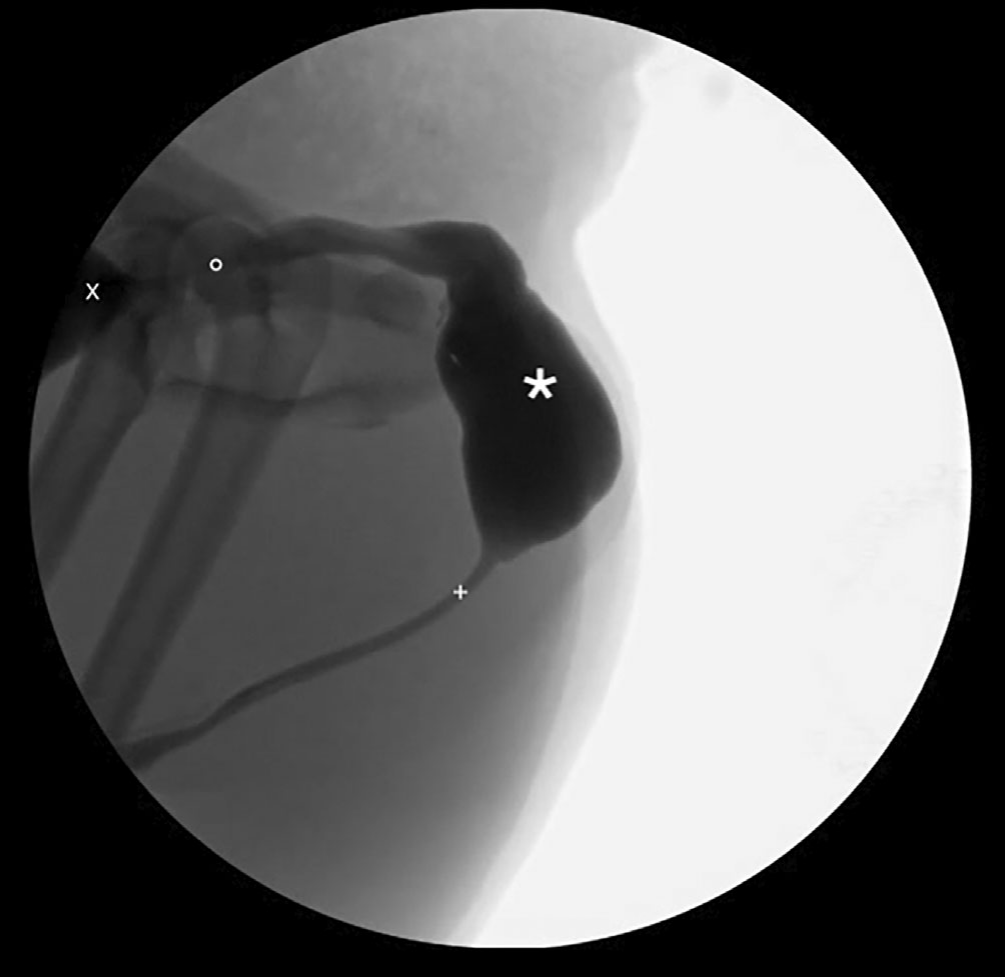
Contrast cystourethrogram performed under fluoroscopic guidance. The dog is placed in lateral recumbency. A saccular urethral diverticulum extending from the prostatic urethra to the membranous urethra is visible (*). Our study was performed before AUS removal because of persistent dysuria. A urethral stricture was diagnosed at the site of the AUS (°). Urinary bladder (X), normal urethra (+).

**FIGURE 6:**
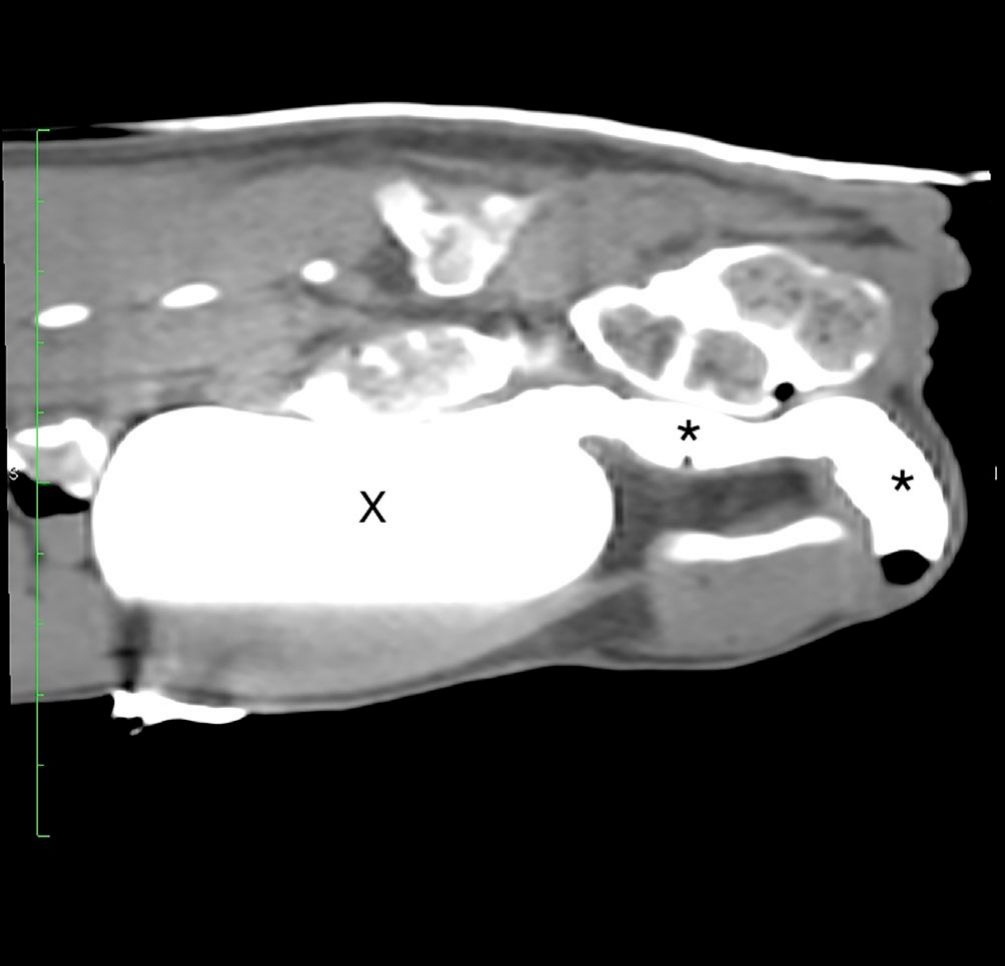
A computed tomodensitometry excretory urography revealing severe urethral dilatation extending from the prostatic urethra to the membranous urethra (*). Urinary bladder (X).

Cystoscopy and retrograde positive contrast cystourethrography under fluoroscopic or radioscopic guidance was performed in 7/8 dogs and confirmed the UD (Figures 1, 2, 3, 4, 5). Contrast CT was diagnostic for the UD in 1/8 dog (Figure 6).

As all diverticula presented a localized pouch‐like structure, they were all classified as saccular with 1 being bilobed. Diverticula were located in the prostatic urethra in 7/8 dogs and in the prostatic and membranous urethra in 1/8 dog.

### 3.4 Surgery

**FIGURE 7:**
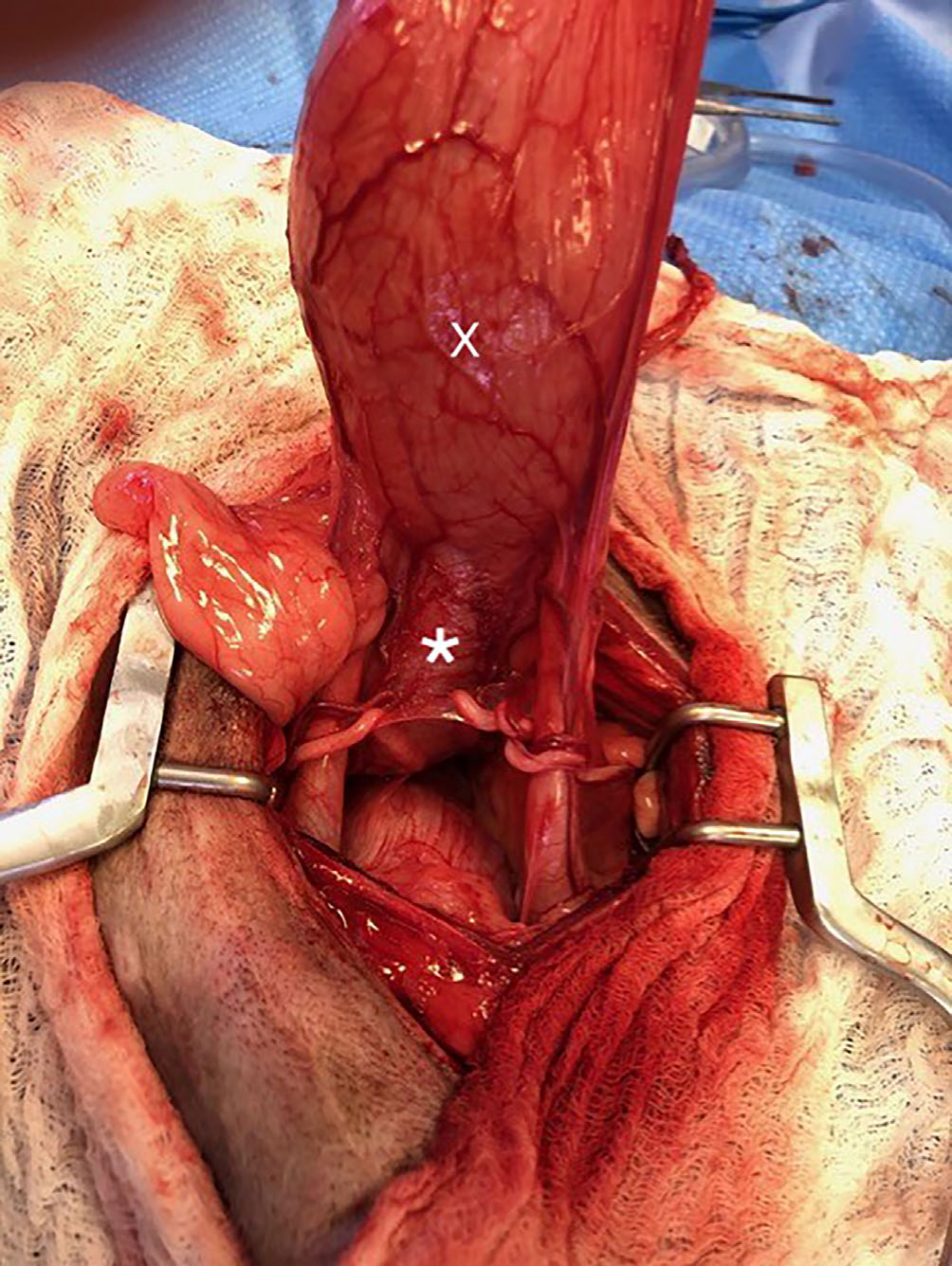
Intraoperative image of the bladder (X) and a dilated prostatic urethra (*) consistent with a proximal saccular urethral diverticulum.

**FIGURE 8:**
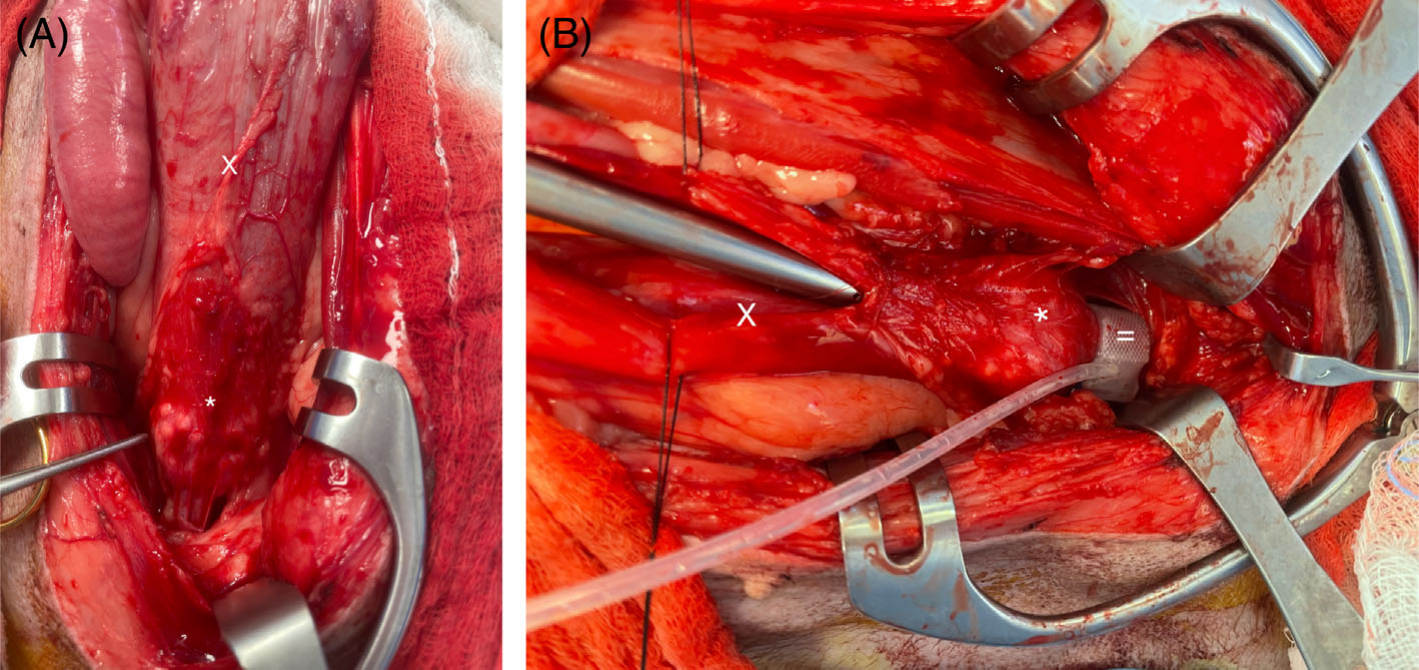
(A) and (B) Intraoperative image of the bladder (X). The prostatic urethra is dilated consistent with a saccular urethral diverticulum (*). An AUS (=) is placed around the prostatic urethra distal to the urethral diverticulum.

Artificial urethral sphincter were surgically placed by a board‐certified surgeon in all dogs. Dissection of the urethra caudal to the prostate was performed by placing traction sutures at the bladder apex and pulling the bladder cranially to access the distal 3rd of the prostatic urethra (Figures 7 and 8A). During the surgery, the prostate was normal in appearance in 3 dogs, hypoplastic in 3 dogs, not visualized in 1 dog, and no mention of the prostate was found in the surgical report in 1 dog. In 1 dog with a hypoplastic prostate, multiple white nodules were also observed on the surface of the prostate. In 6/8 dogs, the AUS was placed distal to the UD in the distal 3rd of the prostatic urethra (Figure 8B). In 1 dog with a bilobed diverticulum, the AUS was placed between the 2 lobes. In the dog with the UD located up to the membranous urethra, the AUS was placed between the bladder trigone and the prostate, thus cranial to the UD (Figure 5). The following cuff sizes were placed: 10 × 14 mm (n = 3), 12 × 14 mm (n = 1), 14 × 14 mm (n = 2) and 16 × 14 mm (n = 2). No complications occurred during placement. Dogs were discharged a median of 2 days (range, 1‐6; mean, 2.25) postoperatively with empty cuffs.

### 3.5 Minor and major complications

Neither intraoperative nor perioperative complications were reported. Two minor complications were reported 1 year after AUS placement in 2/8 dogs, which presented for straining to urinate and urinary incontinence secondary to an overdistended bladder. Deflation of the cuff was sufficient to allow both dogs to urinate with a good stream. One major complication was observed 4.5 years after AUS placement in 1/8 dog. The dog was presented for stranguria and straining to urinate of 6 months duration. The cuff was partially deflated 2 months after the 1st onset of clinical signs. A contrast study after complete deflation of the cuff revealed obstruction at the level of the AUS with marked dilatation of the proximal urethra. A caudal celiotomy revealed that a fibrous capsule had formed around the AUS resulting in partial obstruction of the urethra. Suppurative exudate was observed within the capsule, and *S pseudintermedius* was cultured. The AUS and the fibrous capsule were removed, and the dog was able to urinate.

### 3.6 Follow‐up and outcome

Six of 8 dogs were alive at the time of writing. Persistent incontinence after AUS placement requiring inflation of the cuff occurred in 7/8 dogs. The last dog was almost fully continent after AUS placement, and the owner declined inflation. A 1st cuff inflation was performed a median of 40 days (range, 22‐107; mean, 53.4) after AUS placement. The median number of follow‐ups was 4 (range, 1‐8; mean, 4.13). The median total volume of saline injected in the cuff was 1.2 mL [range, 0.5‐2.4; mean, 1.31].

Overall, AUS placement and cuff inflation resulted in improvement in UCS in all dogs. The median continence score was 4/5. Incontinence was partially improved (UCS 3/5, n = 1; UCS 4/5, n = 5) in 6/8 dogs and resolved (UCS 5/5) in 2 dogs. One of the dogs with a UCS of 5/5 was regularly evaluated for inflations of the cuff and required adjustment of the volume within the cuff periodically to maintain a UCS of 5/5. Scores were attributed based on communications with the owner after cuff inflations.

Six of 8 dogs continued to have regular follow‐ups. The last follow‐up occurred a median of 518 days (range, 82‐781 days) after AUS placement with a median UCS of 4/5. The dog who had his AUS removed had a median UCS of 4/5 at 2359 days after AUS placement. After removal of the AUS, the UCS decreased to 3/5. One dog was lost to follow‐up 63 days after AUS placement and after 2 inflations of the cuff. On last follow‐up, the dog had a UCS of 3/5. A phone recheck 628 days after AUS placement (at time of writing) reported a UCS of 3/5.

Two dogs were euthanized for reasons not related to urinary incontinence. Initially almost fully continent after AUS placement (UCS 4/5 for 2 years), the 1st dog was euthanized 1484 days after surgery because of uncontrolled seizures and poorly controlled diabetes mellitus. Urinary continence score decreased to 1/5 after diagnosis of the diabetes mellitus. The 2nd dog had a UCS of 4/5; however, the score decreased to 1/5 after the administration of prednisone for treatment of suspected inflammatory bowel disease. Cuff inflation resulted in a UCS of 4/5. The dog was euthanized 900 days after AUS placement for severe recurrent epistaxis at the age of 9 years.

## 4 DISCUSSION

In veterinary medicine, literature is scarce regarding UD in male dogs with only 2 cases previously reported.^1^, ^2^ In both cases, a surgical diverticulectomy was performed and resulted in improved but persistent urinary continence in the long term. This multi‐institutional study describes a dog with UD treated with an AUS. Improvement in UCS continence scores was observed in all dogs.

The recent consensus on diagnosis and management of urinary incontinence in dogs consider vesicourethral diverticula as a disorder of storage and voiding.^14^ Two of 8 dogs presented a weak urine stream consistent with a voiding disorder, but 6/8 dogs had a completely normal micturition pattern. A postvoiding residual volume measurement was not performed at the time of diagnosis nor during the follow‐up visits. This diagnostic tool described in the consensus would be indicated to classify the etiology of the urinary incontinence in dogs with UD.

Our study provides a better understanding of the anatomic and clinical presentation of UD in male dogs. Although all the UD were of the saccular type, a wide variety in respect to size and appearance was found. In our study, all UDs were located within the prostatic urethra, and 1 UD extended up to the membranous urethra. Two locations have been defined in humans: anterior (penile and bulbar urethra) and posterior (membranous and prostatic urethra). Anterior UD are more prevalent than posterior UD in humans^15^; a recent study reported a prevalence of 83% and 17% for anterior and posterior UD, respectively, regardless of etiology.^5^

A congenital origin was more common (7/8 dogs) than an acquired UD (1/8). In humans, UD have various underlying causes: congenital, traumatic, iatrogenic, or neoplastic.^5^, ^15^, ^16^ In humans, a congenital etiology is reported in 8% to 33% of the cases and could result from incomplete development of the ventral aspect of the urethra.^5^, ^17^, ^18^ As opposed to the findings in our study, acquired UD in humans is more prevalent than congenital UD and mainly occurs as a complication of previous urologic interventions.^5^, ^16^ Other iatrogenic causes are prolonged catheterization, stricture formation secondary to radiation therapy, or inflammation caused by urethroscopy or other medical instrumentation.^5^, ^15^, ^16^, ^17^ Placement of an AUS is associated with secondary urethral diverticulum in humans.^5^, ^16^, ^19^, ^20^

The reason of initial presentation for all dogs was severe and persistent urinary incontinence. A weak urine stream was reported in 2 dogs. Urinary incontinence (37%), recurrent urinary tract infections (23%), and obstruction of the lower urinary tract (19%) are the 3 clinical signs most reported in the largest study of UD in humans. These clinical signs are believed to occur secondary to urine stasis and obstruction.^5^ Five of 8 dogs had a positive urine culture before the diagnosis of UD, although no dog demonstrated lower urinary tract signs other than incontinence.

In human medicine, 3 therapeutic approaches are described for treatment of UD: surgical diverticulectomy and urethral reconstruction, urinary diversion, and nonsurgical techniques.^5^, ^16^ However, in humans, UD is commonly associated with other forms of urethral anomalies such as stricture and fistula, which also require surgical correction.^4^ Diverticulectomy and surgical reconstruction of the urethra is the most common surgical procedure used in humans, being performed in 42% to 55% of the patients.^5^, ^16^, ^21^ Urine diversion (8%‐13.5% of cases) consists of ileal conduit or suprapubic tube placement and is commonly used in patients with a neurogenic bladder.^5^, ^16^ Nonsurgical techniques (21%‐32% of cases) are usually performed in asymptomatic patients, in patients without urethral obstruction and recurrent urinary tract infection, or in poor surgical candidates.^5^, ^16^

The complications (16.6%‐26%) associated with a diverticulectomy and urethral reconstruction are urethrocutaneous fistula, recurrence of the UD, epidymo‐orchitis, and febrile urinary tract infection.^5^, ^16^, ^21^ Up to 18% of patients that underwent diverticulectomy without postoperative complications continued to present persistent urinary clinical signs.^5^ Persistent urinary incontinence is described in several studies and is generally managed with a penile clamp.^16^, ^17^

In the present study, improvement of the incontinence was observed in all cases. This was also the case for the 2 previous case reports of UD in dogs who underwent surgical management.^1^, ^2^ To compare the results of the 2 case reports and our study, we assigned to the 2 dogs described in the case reports a UCS of 1/5 at presentation. Two years after surgery, the urinary incontinence of 1 dog improved and was consistent with a UCS of 4/5. In the 2nd, a UCS of 5/5 was achieved 3 weeks after surgery; however, 5 weeks after surgery, the dog started dribbling urine and a UCS of 3/5 to 4/5 was assigned.^1^, ^2^ In our study, an improvement of the urinary incontinence was observed in all dogs, and the median continence score increased from 1/5 at presentation to 4/5 after AUS placement. Comorbidities such as diabetes mellitus or prednisone treatment affected urinary continence. A major advantage of the AUS is the ability to adjust the cuff as needed to maintain continence. Artificial urethral sphincter placement also has the advantage of being less invasive compared with diverticulectomy and urethral reconstruction. For AUS placement, the surgical approach and the dissection are centered around the proximal 3rd of the urethra and can be performed through a caudal celiotomy. Traction on the bladder helps expose the urethra and fracture of the pubis to reach the pelvic urethra is not required. Dogs in our study were discharged a median of 2 days (range 1‐6; mean: 2.25) after surgery. In the 2 dogs that underwent diverticulectomy, a standard ventral midline incision was made; significant dissection of the UD and a reconstruction of the urethra was required. Dogs were hospitalized with a urinary catheter and discharged 5 and 6 days after surgery.^1^, ^2^

After surgery complications in the 2 dogs were persistence of multiresistant asymptomatic bacteriuria 2/2 and persistent urethral dilatation 1‐month after surgery in 1 dog.^1^, ^2^ In our study, 3/8 dogs were presented for straining to urinate 1 and 4.5 years after AUS placement, and 1 dog was presented for recurrent urinary incontinence 1 year after AUS placement. In 2 dogs, deflation of the cuff resulted in a normal urine stream, whereas in the other, removal of the AUS was required because of partial urethral obstruction caused by extraluminal compression by a fibrous capsule that encircled the urethra and AUS. This complication is previously reported in dogs after AUS placement.^9^

Our study did not evaluate the consequences of the AUS on the UD. In humans, it has been reported that UD is a potential complication after AUS placement.^5^, ^16^, ^19^, ^20^ Thus, the resistance to urine outflow could increase the pressure within the urethra and lead to the formation of a UD. The placement of an AUS in a dog with preexisting UD could exacerbate the urethral diverticulum over time, with outcomes that are currently unknown. Subsequent evaluations using contrast urethrography might have provided insights into the progression of this condition.

A recent study assessing the outcome of AUS placement in male dogs with urethral sphincter mechanism incompetence without a UD reported that 16/19 (84%) dogs had an improvement in their continence score and 13/19 dogs (68%) became fully continent within 12 months. Long‐term (>12 months, median 1785 days after AUS placement) urinary incontinence improved in 9/15 (60%) dogs; however, only 8/15 (53%) dogs remained fully continent.^7^ Among these 15 dogs, 1 was euthanized, and in 2 dogs, the AUS was removed because of complications. Our study used the same UCS system as the current study and median UCS preoperatively (n = 19 dogs), in the short‐term (n = 18 dogs) and long‐term periods (n = 12 dogs) were 1/5, 5/5, and 5/5, respectively.^7^ In the present study, 100% of the dogs with UD treated with an AUS had an improvement in their UCS with a median UCS score of 4/5. However, only 2 dogs became fully continent in the long term, and 1 of them necessitated regular inflation of the cuff to maintain a UCS of 5/5. All dogs had at least 1 inflation of their cuff except 1 dog that had a marked improvement (estimated at 95% according to the owner) of his urinary incontinence (UCS 1/5 at presentation and 4/5 after AUS placement), and the cuff was therefore not inflated. Male dogs with UD in our study achieved poorer UCS after AUS placement compared with male dogs with urethral sphincter mechanism incompetence without UD that underwent AUS placement. This is not surprising given the severity of the UD in the dogs in our study.

Concurrent urogenital anomalies were observed in 7/8 dogs presented with UD: ultrasound changes consistent with chronic pyelonephritis or renal dysplasia, cryptorchidism, and pelvic bladder were the 3 most common anomalies. The dog in the diverticulectomy case report was also cryptorchid.^1^ The relationship between UD and cryptorchidism merits further investigation. A urine culture was performed in all dogs and was positive in 5/8 before diagnosis of the UD. Urine cultures performed after AUS placement were negative. Urinary incontinence and pooling of urine within the UD could predispose dogs to bacteriuria. Artificial urethral sphincter placement might have limited urine pooling and decreased the likelihood of a positive urine culture. The prostate was hypoplastic (n = 3/8) or not visualized in 1/8 dogs during the surgical procedure. Interestingly, in a previous report, the prostate was also not visible.^2^ Thus, a prostatic aplasia or an absence of the prostate could also be associated with congenital UD. A thorough urogenital examination, including urine culture and urogenital ultrasound, is recommended by the authors in dogs diagnosed with a UD.

In humans, UD are diagnosed by retrograde urethrography, voiding cystourethrogram, magnetic resonance imaging, cystoscopy, intraoperative evaluation, urodynamic studies, and physical examination. Multiple imaging studies are usually performed to diagnose and characterize the UD: cystoscopy and contrast imaging being the most commonly used.^5^ In our study, cystoscopy and cystourethrography were combined and diagnostic in 7/8 dogs. In 1 dog, abdominal ultrasonography was 1st performed and revealed a tortuous and dilated proximal urethra. The final diagnosis of UD was confirmed by a contrast CT. In all cases, a contrast study allowed visualization and localization of the UD. Cystoscopy to rule out ureteral ectopia before AUS placement is recommended by the authors. Unlike in humans, in whom swelling on the ventral aspect of the penile urethra or a penoscrotal mass can be palpated, physical examination and rectal palpation were not helpful in identifying UD in the dogs in our study.^5^, ^16^, ^22^

Our study has several limitations. The main limitation is the retrospective nature and lack of standardization particularly regarding diagnostic work‐up and timing of rechecks. Initial medical management, before AUS placement, was not consistent, and a standardized continence scale was not used to assess incontinence before and after surgery and long term. Urinary continence score was attributed retrospectively based on information in the medical files and after client communications. The main difficulty was attributing retrospective UCS differentiating a score of 3/5 and a score of 4/5 because of a lack of information in the medical file. Although an improvement in all dogs was reported after AUS placement, the median continence score could have been over or underestimated. A standardized set of questions for the owners at the time of presentation and upon rechecks could have more accurately assessed UCS in each dog.

Despite a broad recruitment across various groups of veterinary specialists most likely to encounter these dogs, only 8 dogs with UD treated with an AUS were recruited. The authors believe that, given the severity of UD in some dogs and absence of publications reporting response to AUS placement, this therapeutic option is not offered to owners.

In conclusion, UD is an anomaly that should be considered in male dogs presented with urinary incontinence. Placement of an AUS was an effective treatment for urinary incontinence secondary to UD in male dogs. Although the number of cases in our study is limited, placement of an AUS resulted in long‐term improvement in continence. Based on the outcome of this small study, an AUS should be considered for treatment of urinary incontinence in dogs diagnosed with UD.

## CONFLICT OF INTEREST DECLARATION

Authors declare no conflict of interest.

## OFF‐LABEL ANTIMICROBIAL DECLARATION

Authors declare no off‐label use of antimicrobials.

## INSTITUTIONAL ANIMAL CARE AND USE COMMITTEE (IACUC) OR OTHER APPROVAL DECLARATION

Authors declare no IACUC or other approval was needed.

## HUMAN ETHICS APPROVAL DECLARATION

Authors declare human ethics approval was not needed for this study.
